# Implementing a Novel Machine Learning System for Nutrition Education in Diabetes Mellitus Nutritional Clinic: Predicting 1-Year Blood Glucose Control

**DOI:** 10.3390/bioengineering10101139

**Published:** 2023-09-28

**Authors:** Mei-Yuan Liu, Chung-Feng Liu, Tzu-Chi Lin, Yu-Shan Ma

**Affiliations:** 1 Department of Nutrition, Chi Mei Medical Center, Tainan 710402, Taiwan; m880419@mail.chimei.org.tw; 2 Department of Nutrition and Health Sciences, Chia Nan University of Pharmacy & Science, Tainan 710402, Taiwan; 3 Department of Food Nutrition, Chung Hwa University of Medical Technology, Tainan 710402, Taiwan; 4 Department of Medical Research, Chi Mei Medical Center, Tainan 710402, Taiwan; yushan.ma.72@gmail.com; 5 Nursing Department, Chi Mei Medical Center, Liouying, Tainan 73657, Taiwan

**Keywords:** diabetes mellitus (DM), machine learning, artificial intelligence, feature importance, predictive system, glycosylated hemoglobin (HbA1c), well-controlled HbA1c, diabetes-related disease, nutrition education

## Abstract

(1) Background: Persistent hyperglycemia in diabetes mellitus (DM) increases the risk of death and causes cardiovascular disease (CVD), resulting in significant social and economic costs. This study used a machine learning (ML) technique to build prediction models with the factors of lifestyle, medication compliance, and self-control in eating habits and then implemented a predictive system based on the best model to forecast whether blood glucose can be well-controlled within 1 year in diabetic patients attending a DM nutritional clinic. (2) Methods: Data were collected from outpatients aged 20 years or older with type 2 DM who received nutrition education in Chi Mei Medical Center. Multiple ML algorithms were used to build the predictive models. (3) Results: The predictive models achieved accuracies ranging from 0.611 to 0.690. The XGBoost model with the highest area under the curve (AUC) of 0.738 was regarded as the best and used for the predictive system implementation. SHAP analysis was performed to interpret the feature importance in the best model. The predictive system, evaluated by dietitians, received positive feedback as a beneficial tool for diabetes nutrition consultations. (4) Conclusions: The ML prediction model provides a promising approach for diabetes nutrition consultations to maintain good long-term blood glucose control, reduce diabetes-related complications, and enhance the quality of medical care.

## 1. Introduction

Type 2 diabetes mellitus (T2DM) is a significant public health concern, placing a substantial burden on human life and health. It not only affects an individual’s quality of life but also increases the risk of mortality and complications such as cardiovascular disease, cerebrovascular disease, diabetic nephropathy, retinopathy-induced blindness, and peripheral vascular neuropathy leading to amputation. These complications impose substantial social and economic costs [1]. Managing T2DM requires ongoing interventions, including nutritional therapy, exercise routines, medication management, self-care practices, psychological support, and smoking cessation [2]. Nutrition education plays a crucial role in the long-term management of diabetes, involving discussions, assessments, lifestyle adjustments, and ongoing monitoring for complications [3]. With guidance from a medical team, lifestyle changes and self-care knowledge taught by educators can contribute to improved prognosis, health conditions, and quality of life for patients [4].

HbA1c (glycated hemoglobin) reflects an individual’s blood sugar fluctuations over the past three months before measurement and serves as an essential predictor of diabetes complications. It helps assess whether patients and their treatment are achieving or maintaining glycemic control goals [5]. HbA1c control within the first year of diabetes diagnosis strongly correlates with the occurrence of major and minor vascular diseases and mortality ten years later [6]. Accurately predicting whether a patient’s HbA1c level can be less than 7% (well-controlled) within one year after the primary diagnosis can greatly assist in tailoring a long-term nutritional care plan for the patient. This approach aligns with the principles of personalized medicine and precision medicine advocated in recent years [7]. However, currently, no available tool offers personalized and accurate long-term predictions for diabetes. Recent advancements in machine learning (ML) algorithms and computing speed present an opportunity to address this gap using artificial intelligence (AI)/ML technology.

In Taiwan, the National Health Insurance Administration (NHIA) implemented a regulation in 1995 that facilitated the rapid sharing of medical information across hospitals. In 2001, the government introduced the pay-for-performance program [8], which enables the systematic monitoring and treatment of diabetic patients over an extended period. Chi Mei Medical Center, as one of Taiwan’s largest hospitals, has accumulated extensive data on diabetes treatment over the past 13 years, including comprehensive records of dietitian interventions and outcomes.

In this study, we leveraged this big medical data to develop an AI system that predicts whether HbA1c levels can be well-controlled below 7% within a year after the initial diabetes diagnosis because an HbA1c level with a value of 7% is regarded as a well-controlled HbA1c level in practice [5,9]. We identified feature variables based on the medical literature and expert clinical experience. AI models often have complex nonlinear or network structures, presenting challenges in terms of interpretability. That is, explainable AI (XAI) is needed during AI development [10]. To address this, we utilized SHAP (SHapley Additive exPlanations) analysis [11], a method of XAI, to visually demonstrate the importance of each feature variable in the built prediction model.

Our AI prediction system empowers clinical health educators to understand and predict changes in HbA1c levels for diabetic patients based on their current physiological statuses. It serves as a valuable reference for clinical care and nutrition education interventions, enhancing patients’ disease awareness, reinforcing the importance of lifestyle changes, and motivating positive behavioral modifications. Furthermore, by considering the AI prediction results, medical teams can intervene early; supplement their advice regarding medications, disease-specific diets, and exercise requirements; promote shared decision making; and improve the quality of medical care.

In the past, most AI/ML studies have centered on evaluating model quality, with only a limited number of predictive systems developed for medical condition prognosis [12,13,14]. Furthermore, predictive systems in the realm of nutrition and healthcare AI remain sparse [15]. Consequently, this study significantly adds to the advancement in this domain.

## 2. Materials and Methods

### 2.1. Research Design

**Figure 1:**
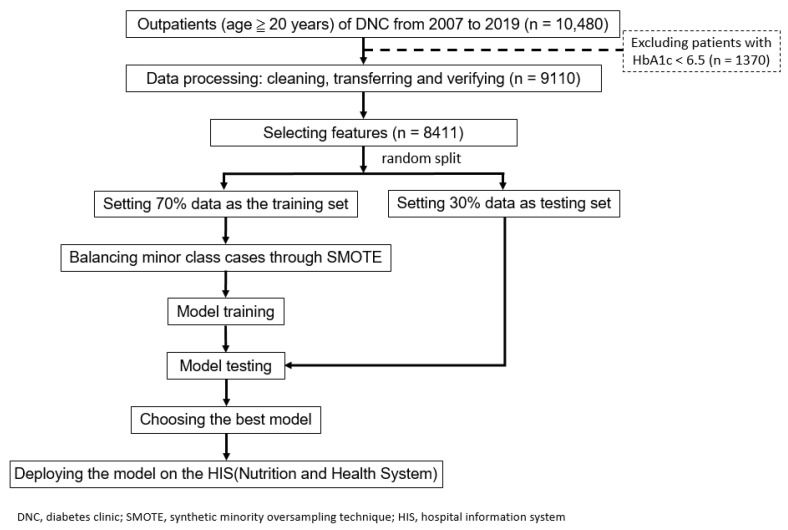
Research flowchart.

In this research, we sought to develop AI models that predict whether an individual outpatient with T2DM can maintain HbA1c levels below 7% within a year of their initial diabetes diagnosis. We identified feature variables based on the medical literature and expert clinical experience. This retrospective study received approval from the institutional review board of Chi Mei Medical Center (no. 10901-014). To ensure the protection of patient privacy, all patient data were de-identified. As this study is retrospective, the need for informed consent from the patients was waived. The flowchart outlining the study process is presented in Figure 1.

### 2.2. Setting

The data for this study were obtained from the Nutrition Education System Database of Chi Mei Medical Center in Taiwan. This study included T2DM outpatients, including those with gestational diabetes and those aged 20 years and older who participated in the pay-for-performance (P4P) program and received health education in the diabetes nutrition clinic from 2007 to the end of 2019. We ensured that there was no selective inclusion of participants, thus maintaining fairness and avoiding selection bias. Patients with a current HbA1c level of below 6.5% were excluded from this study. A total of 8411 patients were enrolled.

### 2.3. Definition of the Model’s Outcome Variable

Maintaining HbA1c levels below 7% is clinically regarded as well-controlled blood glucose in DM patients [5,9]. Thus, we decided to set the cutoff threshold at 7% as the target to predict, with the binary outcome variable coded ‘1’ for maintaining HbA1c levels below 7% or less after one year, and coded ‘0’, otherwise. Patients whose current HbA1c levels were below 6.5, indicating not being diagnosed as DM, were excluded.

### 2.4. Feature Variables and Selection

A total of 18 feature variables, or impact factors, were proposed based on the relevant medical literature [16,17,18,19,20] and expert clinical experience. These variables included demographic information (age, gender, BMI, and length of illness), physical activity (exercise or no exercise), dietary intake (daily calories, average meals per day, protein, lipids, and carbohydrates), and blood biochemistry values (fasting blood glucose (glucose AC), HbA1c, total cholesterol, triglycerides (TGs), LDL cholesterol, HDL cholesterol, C-reactive protein (CRP), and estimated glomerular filtration rate (eGFR)). The feature “length of illness” denoted the duration for which a patient had been afflicted with diabetes prior to their first visit to our diabetes outpatient clinic.

### 2.5. Data Preprocessing and Machine Learning Modeling

The required data were extracted from the outpatient diabetic nutrition counseling system, and data with ambiguous values were checked and corrected. We observed that the pattern of missing data was consistent and appeared to be random, with each feature having a missing ratio of less than 4%. Thus, we opted to exclude the missing data without resorting to any imputation techniques. The dataset was divided into a training set (70% of the data) and a validation set (30% of the data) for model training and evaluation, respectively. Accuracy, sensitivity, specificity, and the area under the curve (AUC) were used as evaluation metrics. Prior to the model training, the training set underwent preprocessing to address data imbalances in the positive outcome using the synthetic minority over-sampling technique (SMOTE) [21]. Five supervised machine learning algorithms, including logistic regression (LR), random forest (RF), multilayer perceptron (MLP), light gradient boosting machine (Light GBM), and extreme gradient boosting (XGBoost), were used to build the models.

### 2.6. Prediction System Implementation and Trial Use

The best model was determined based on the AUC values, and the information technology engineers implemented the model into a prediction system for trial use by dietitians. The model was built using the Python programming language with the scikit-learn machine learning library, while the web-based user interface was created using MS Visual Studio® software (v 17.7). Both components were then integrated into an AI prediction system aimed at supporting nutrition education.

## 3. Results

### 3.1. Basic Case Information and Lifestyle Analysis

After excluding missing values, a total of 8411 patients from the diabetes nutrition clinic (DNC) at Chi Mei Medical Center were included in the machine learning model.

An analysis of basic information and daily living habits revealed 3171 patients with HbA1C levels below 7% within one year (37.7%) of their first visit, and 5240 otherwise (37.7%). There were significantly higher trends in age and average meals per day in the <7% group, indicating that older patients had a greater chance of maintaining their HbA1c levels after one year. Meanwhile, in comparison, patients with HbA1c levels greater than 7% after one year exhibited longer lengths of illness and significantly lower trends in exercise, cho. total, TG, glucose AC, and current HbA1c levels. The features of gender, BMI, protein, lipids, carbohydrates, daily calories, cho. LDL, cho. HDL, eGFR, and CRP/hs-CRP did not show significant differences between the two groups. The details are summarized in Table 1.

### 3.2. Analysis of Blood Biochemistry Results

As shown in Table 1, the blood biochemical values determined were 167.1 ± 70.8 mg/dL for fasting blood glucose, 114.0 ± 39.7 mg/dL for LDL cholesterol, 47.6 ± 13.5 mg/dL for HDL cholesterol, 187.9 ± 47.3 mg/dL for total cholesterol, 169.0 ± 171.9 mg/dL for TGs, 9.0 ± 2.3% for current glycosylated hemoglobin (HbA1c), and 72.8 ± 22.8 for the estimated glomerular filtration rate (e-GFR). Moreover, Spearman’s correlation analysis identified the correlation between the outcome and each feature variable (Table 2). It revealed that length of illness and current HbA1c levels had the highest correlations with the outcome, while gender, carbohydrates, and eGFR had the lowest correlations.

### 3.3. Prediction Model Building and Feature Importance Analysis

In this study, several common and advanced machine learning algorithms were employed to predict whether patients would control HbA1c levels below 7% after one year using the 18 feature variables. The algorithms used included logistic regression (LR), random forest (RF), multilayer perceptron (MLP), light gradient boosting machine (light GBM), and extreme gradient boosting (XGBoost). A grid search with five-fold cross-validation for hyperparameter (Table 3) tuning for each algorithm was conducted to obtain the optimal model.

The accuracy of the prediction methods ranged from 0.611 to 0.690. Among these algorithms, XGBoost demonstrated the highest accuracy of 0.690, sensitivity of 0.684, specificity of 0.693, and an area under the curve (AUC) value of 0.738. The sensitivity, specificity, and AUC values for all the algorithms are presented in Table 4.

**Figure 2:**
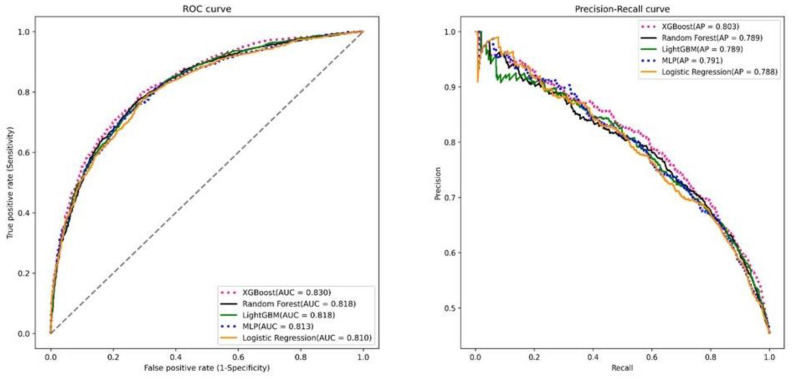
The receiver operating characteristic curves and the precision–recall curve.

To visualize the results, the receiver operating characteristic (ROC) curves and the precision–recall curves were plotted, as shown in Figure 2 These curves provide graphical representations of the performances of the prediction models and their abilities to discriminate between positive and negative outcomes. Overall, the XGBoost algorithm was identified as the best prediction model in terms of accuracy, sensitivity, specificity, and AUC. The ROC curves and precision–recall curves provide additional insights into the performances of the models and their potential usefulness in predicting HbA1c reduction after one year. We performed the DelongTest to compare the model qualities. The results show that there was no significant difference between the LightGBM model and the XGBoost model, but the remaining models were significantly different from the XGBoost model.

**Figure 3:**
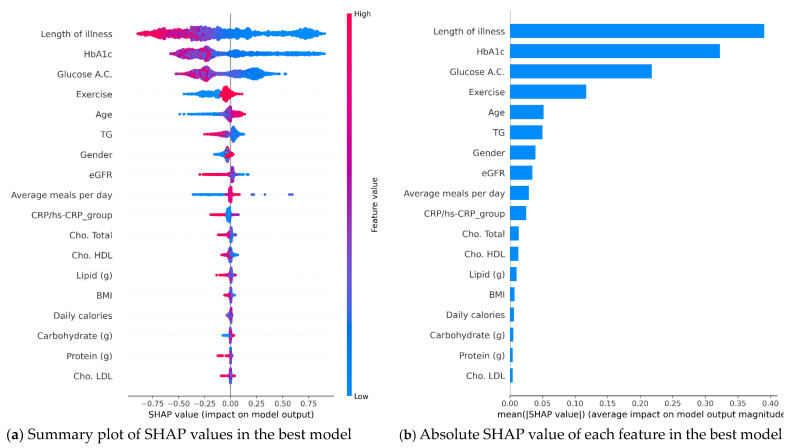
The feature importance plots of SHAP analysis.

Furthermore, we conducted a SHAP analysis for feature importance to interpret how each feature contributed to the prediction in a visual manner. A SHAP value of >0 means that it is positively related to the outcome, and vice versa. For example, in Figure 3a, the smaller the length of illness one year later, the higher the probability of controlling HBA1 below 7%, and patients with exercise habits have a higher chance of having HBA1 levels of <7 one year later. This analysis helps us understand why certain features were considered more or less important in the best XGBoost model. The feature importance plot shown in Figure 3 allows us to identify the order of importance of the model features. According to the feature importance plot of the XGBoost model (Figure 3b), we can clearly observe that the top three influential factors in the best model for predicting 1-year HbA1c levels of <7% are the length of illness, current HbA1c levels, and glucose AC.

### 3.4. Prediction System Implementation and User’s Acceptance

**Figure 4:**
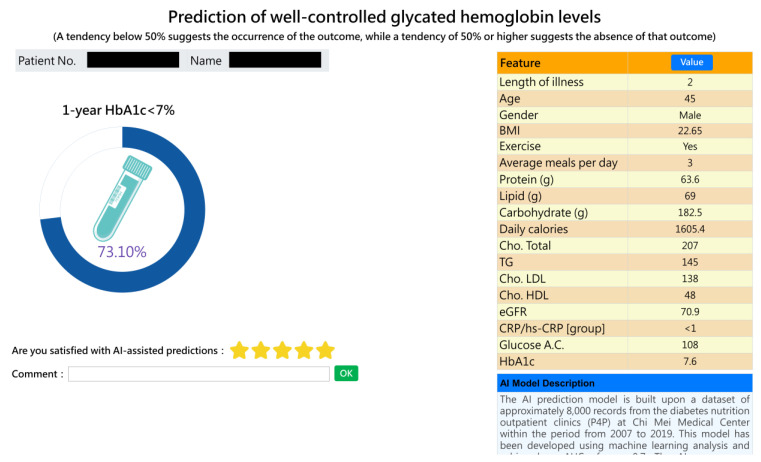
Prediction system picture.

The best model was successfully implemented in a web-based forecasting system. The system screen, as depicted in Figure 4, displays the graphical interface and user-friendly design of the prediction system. This visual representation of predictions adds value by providing a clear and intuitive understanding of the patient’s expected outcomes. At present, the AI prediction system has been integrated into the workflow of dietitians and provides real-time and automatic prediction without manual input. Overall, the feedback received from the dietitians indicates positive acceptance and appreciation of the prediction system. The system’s graphical interface and specific prediction rates were identified as valuable tools for personalized patient care and effective communication within the medical team.

Seven nutritionists were given the opportunity to use the system and provide feedback. We collected and analyzed their experiences and suggestions to assess user acceptance of the system. We asked three structured questions (on a five-point scale, one point indicating strongly disagree, and five points strongly agree): (1) Is it easy to operate? (2) Is it clinically useful? (3) Are you willing to use it? They were also encouraged to provide other comments. The survey results show that they were positive about the prediction system (the mean values of ease-of-use, usefulness, and use intention were 4.4, 3.9, and 4.1, respectively), but the score for usefulness was only 3.9, showing that the nutritionists were still not very satisfied with the system’s functions. Moreover, they expressed particular appreciation for the graphical interface, which provides specific prediction rates that allow for personalized and accurate predictions of potential improvement in a patient’s condition. The dietitians found that the tool could improve communication between the healthcare team and patients, facilitating discussions about subsequent nutritional treatment plans.

### 3.5. Comparative Models Utilizing Alternative Feature Selection Methods

We conducted a comparison between the best all-feature model (18 features) and the significant-feature model (8 features, as indicated in Table 1) using the DeLong test. As illustrated in Table 5, although the all-feature model exhibited a slightly better performance compared with the significant-feature model, the difference did not reach statistical significance (*p* = 0.085). This suggests that the significant-feature model could be considered for clinical use, particularly when healthcare resources are limited.

## 4. Discussion

The use of AI models to develop a chronic disease nutritional status monitoring system for assessing prognostic risk is an area that lacks extensive research. However, there have been some studies exploring the use of machine learning techniques to predict the individual risk of cardiometabolic disease based on dietary or supplement intake. One such study by Panaretos et al. [22] utilized the KNN algorithm and RF decision tree to evaluate cardiometabolic risk over a 10-year period. They found that these AI/ML techniques explained a significant portion of the cardiometabolic risk, with the RF decision tree outperforming the KNN algorithm. The study also highlighted the advantages of machine learning techniques over logistic regression classification for predicting health disease risk.

The present study aligns with this research trend and contributes to it by building the best model, specifically an XGBoost-based model, which surpasses the results obtained by Panaretos et al. This study is, to the best of our knowledge, the first implementation study to utilize AI/ML technologies to predict the control of changes in HbA1c levels after one year in patients with diabetes and successfully apply it in clinical practice. By leveraging the power of AI/ML, this study expands the possibilities for personalized medicine and the use of AI in improving patient outcomes in diabetes management [23].

To explore the model’s explainability, a feature importance plot was generated, revealing 12 prominent factors in the best XGBoost model. Notably, some of these factors, including current HbA1c levels, age, BMI, HDL, and eGFR, were also identified as leading factors in other models such as RF, LR, and Light GBM. This information empowers dietitians to provide targeted recommendations to patients, aiming to strengthen positive factors and mitigate negative factors, thereby increasing the likelihood of long-term reductions in HbA1c levels [24].

Based on the important features identified, we can modify them in our AI prediction system to simulate probability changes and elucidate them to patients, a process known as shared decision making (SDM). However, it is pivotal to recognize that while some elements like exercise and average meals per day can be altered through lifestyle modifications, inherent factors like age and gender remain immutable. For example, a dietitian can illustrate to a specific patient how altering the exercise feature from “No” to “Yes” can shift the probability from 45% (indicating a tendency to not achieve an HbA1c level of <7%) to 56% (indicating a tendency to achieve an HbA1c level of <7%). This visualization can motivate the incorporation of regular exercise routines, such as partaking in physical activities at least thrice a week. By concentrating on adaptable significant factors and offering tailored advice, dietitians can aid patients in effectuating substantive lifestyle modifications and enhancing long-term glycemic control.

In recent years, the digitization of medical data has revolutionized healthcare by enabling clinicians to access vast amounts of historical medical data and develop accurate predictive models for clinical decision making. This predictive tool can also be utilized by healthcare professionals to provide patients with a more precise understanding of their future outcomes, allowing them to actively participate in the decision-making process and improving communication between patients and doctors [19]. This, in turn, enhances patients’ confidence in implementing the recommended changes [7].

The AI model developed in this study has been integrated into the existing DNC information system. As a result, when dietitians collect data on patients’ diets, lifestyles, medication intakes, and nutritional assessments during consultations, they can utilize the predictive model seamlessly without the need for manual input. The model automatically processes the collected data to estimate the patient’s HbA1c improvement one year later. This streamlined approach enables dietitians to provide timely interventions and personalized guidance on diet and lifestyle modifications, fostering effective communication between clinicians and patients in outpatient clinics [25,26,27,28].

In clinical practice, we set blood sugar control goals based on a patient’s condition. Factors such as pre-meal and post-meal blood sugar levels, HbA1c values, age, and the patient’s motivation to improve diabetes through lifestyle changes are all taken into account when predicting their HbA1C reduction target for the following year. With this AI prediction tool, we can assess the likelihood of achieving those goals and adjust nutritional or therapeutic plans accordingly [3]. For instance, for patients who are very likely to have their blood sugar controlled to HbA1c levels of <7% a year later (with a predicted probability of ≥50%), we intensify health education on significant features like exercise and dietary habits. We encourage them to maintain good dietary and living habits once they are back home. For patients with a tougher challenge of controlling their HbA1c levels to <7% a year later (with a predicted probability of <50%) who may struggle with consistent lifestyle and dietary habits, nutritionists not only provide active nutritional education but might also need to discuss with the attending physician about adjusting medication timings and treatment modalities. Overall, this AI prediction system serves as a smart and useful tool to achieve shared decision making between healthcare professionals and patients.

Overall, the AI system in this study stands as a pivotal tool to enhance patient awareness and motivate lifestyle alterations for optimized blood glucose control. It aids in mitigating the risks associated with both macrovascular and microvascular complications by maintaining stable glucose levels [9], ultimately serving as a facilitator in shared decision-making processes between healthcare providers and patients [29].

We recommend that practitioners integrate AI predictive models into the routine care of diabetic patients to identify high-risk individuals early and tailor interventions more effectively. Medical institutions should utilize such models to optimize resource allocation and enhance healthcare delivery, requiring proper training for practitioners in using these models. Regarding policy, it is essential to formulate and implement strategies that integrate AI technologies into healthcare protocols, advocating for the utilization of advanced technologies like IoT and wearables for real-time data acquisition and monitoring, thus improving overall disease management and mitigating the risks of complications.

## 5. Conclusions

In conclusion, our AI prediction system, utilizing the valuable big data accumulated at Chi Mei Medical Center, presents a novel approach for predicting a patient’s 1-year HbA1c change and aiding nutritionists in making informed decisions regarding appropriate nutritional interventions. The system holds significant potential for establishing a personalized health education system, facilitating shared decision making, and enhancing the effectiveness of diabetes nutrition counseling and health education. The feature importance analysis provided a clear understanding of each feature’s impact on the prediction outcome, contributing to the system’s transparency and interpretability.

Furthermore, this study represents an innovative application of AI/ML technology in healthcare practice, particularly in investigating diabetic dietary habits and long-term glycemic control. It aligns with the principles of personalized precision medicine and carries substantial clinical value. We firmly believe that our prediction system can contribute to improving long-term glycemic control, reducing the incidence of diabetes-related complications, and enhancing the overall quality of medical care.

Though patients in this study were enrolled in the P4P program, the results of this study are also applicable to non-P4P patients. However, patients not enrolled in the P4P program receive fewer long-term case management follow-ups and reminders. As a consequence, their disease awareness and adherence to medical instructions may be reduced, which could subsequently impact their chances of improving their HbA1c levels.

Despite the rigorous procedure followed in this study, certain limitations should be acknowledged. Firstly, this study relied on retrospective data from a single medical center in Taiwan, potentially limiting the generalizability of the findings. Additionally, the sample was restricted to patients who participated in the P4P project, and the authenticity of nutrition counseling records, primarily relying on questions asked by medical staff and self-reported patient data, may be challenging to verify. Finally, patients’ varying opinions and responses to nutrition education questions may have introduced common method bias.

Based on our results, we propose several future research directions. Firstly, the effect of medication on predictive models is an interesting but complex research topic that deserves further exploration. Secondly, gathering new patient records, referred to as a testing dataset, is valuable for estimating the expected accuracy of the proposed models to ensure generalizability. Thirdly, expanding the model’s applicability and value by including patients and healthy individuals in the analysis would be beneficial. Fourthly, investigating diabetic outpatients with cardiovascular disease, cerebrovascular disease, diabetic nephropathy, and other related complications could yield valuable insights. Fifthly, considering the long-term impact of diabetes health interventions, incorporating time-series AI algorithms such as RNN and LSTM to develop long-term (multi-year) prediction models holds promise. Sixthly, prospective studies can be designed to explore patients’ compliance with lifestyle changes using AI approaches [30,31]. Lastly, for real-time and continuous prediction, embracing the IoT, wearable technology, and smart technology to directly capture physiological data and daily life records (e.g., diet photos for calorie in-take determination and continuous glucose monitors (CGMs)) from patients through wearable devices and mobile apps would be a valuable avenue to pursue. However, considerations of stability, seamless connectivity, privacy, security, user-friendly interfaces, and affordability are crucial.

## Figures and Tables

**Table 1:** Demographics and feature significance.

Variable	Overall (n = 8411)	One Year Later, HbA1c Level is Greater Than or Equal to 7 (62.3%, n = 5240)	One Year Later, HbA1c Level is Less Than 7 (37.7%, n = 3171)	*p*-Value *
Length of illness, mean (SD)	7.1 (7.3)	8.2 (7.4)	5.2 (6.7)	<0.001
Age, mean (SD)	59.5 (12.2)	59.1 (12.1)	60.0 (12.2)	0.001
Gender, n (%)				
Female	3933 (46.8)	2466 (47.1)	1467 (46.3)	0.491
Male	4478 (53.2)	2774 (52.9)	1704 (53.7)	
BMI, mean (SD)	26.0 (4.3)	26.1 (4.4)	25.9 (4.2)	0.100
Exercise, n (%)				
No	4098 (48.7)	2662 (50.8)	1436 (45.3)	<0.001
Yes	4313 (51.3)	2578 (49.2)	1735 (54.7)	
Average meals per day, mean (SD)	2.7 (1.1)	2.7 (1.2)	2.8 (1.1)	0.004
Protein (g), mean (SD)	61.9 (23.8)	62.0 (21.7)	61.7 (26.8)	0.669
Lipids (g), mean (SD)	61.4 (26.6)	61.7 (24.8)	60.9 (29.3)	0.206
Carbohydrates (g), mean (SD)	191.0 (62.8)	190.7 (62.1)	191.4 (64.0)	0.652
Daily calories, mean (SD)	1602.1 (559.4)	1607.9 (554.1)	1592.5 (568.2)	0.224
Cho. total, mean (SD)	187.9 (47.3)	189.5 (47.6)	185.3 (46.7)	<0.001
TG, mean (SD)	169.0 (171.9)	174.9 (174.2)	159.3 (167.5)	<0.001
Cho. LDL, mean (SD)	114.0 (39.7)	114.6 (39.9)	112.9 (39.4)	0.056
Cho. HDL, mean (SD)	47.6 (13.5)	47.8 (13.7)	47.3 (13.2)	0.136
eGFR, mean (SD)	72.8 (22.8)	72.9 (23.4)	72.7 (21.8)	0.685
CRP/hs-CRP_group, n (%)				
<1	6698 (79.6)	4148 (79.2)	2550 (80.4)	0.335
1 ≤ CRP ≤ 10	772 (9.2)	487 (9.3)	285 (9.0)	
>10	941 (11.2)	605 (11.5)	336 (10.6)	
Glucose AC, mean (SD)	167.1 (70.8)	175.3 (72.4)	153.5 (66.0)	<0.001
HbA1c, mean (SD)	9.0 (2.3)	9.3 (2.2)	8.6 (2.3)	<0.001

Note: * For an alpha level of 0.05, categorical variables (gender, exercise, and CRP/hs-CRP) were evaluated using the chi-squared test approach, whereas numerical features were assessed using the two-sample t-test approach.

**Table 2:** Spearman’s correlations between each feature and outcome (1-year HbA1c levels < 7).

Feature	Correlation Coefficient
Length of illness	−0.244
Age	0.031
Gender	0.008
BMI	−0.016
Exercise	0.053
Average meals per day	0.022
Protein (g)	−0.010
Lipids (g)	−0.016
Carbohydrates (g)	0.005
Daily calories	−0.011
Cho. total	−0.047
TG	−0.059
Cho. LDL	−0.021
Cho. HDL	−0.011
eGFR	0.002
CRP/hs-CRP_group	−0.016
Glucose AC	−0.198
HbA1c	−0.215

**Table 3:** Hyperparameter range for experiments.

Method and Hyperparameter	Value
XGBoost	
learning_rate	1e-3, 1e-2, 1e-1
gamma	0, 1e-2, 1e-3, 1e-4, 1e-5
n_estimators	200, 500, 750, 900, 1000
max_depth	3, 15, 25, 30, 50
num_parallel_tree	2, 5, 15
random_state	8, 16, 29, 42
objective	binary:logistic
LightGBM	
learning_rate	1e-3, 1e-2, 1e-1
n_estimators	120, 200, 500, 750, 1000
max_depth	7, 9, 15, 30, 50, 100
random_state	8, 16, 30, 42
Random forest	
n_estimators	110, 250, 500, 750, 950, 1000
max_depth	7, 9, 15, 30, 45, 50, 100
min_samples_split	2, 5, 10, 15
max_features	auto, sqrt, 0.5, 1.0, 1.5, 2.5
random_state	8, 16, 30, 42
MLP	
hidden_layer_sizes	(125), (125, 35), (100, 75, 30), (100, 55), (100, 75), (100, 45), (100), (96), (90, 60), (90)
max_iter	1000, 500, 250, 200, 100, 50, 30
learning_rate_init	1e-3, 1e-2, 1e-1
early_stopping	True, False
Logistic regression	
penalty	l1, l2
C	np.logspace (−3, 3, 7), 1, 5, 10
max_iter	7, 9, 10, 15, 50, 75, 100

Note: The hyperparameters that are not described in this table are set to the default values used in the scikit-learn library.

**Table 4:** Performance comparison of the machine learning methods (using the XGBoost model as a basis).

Algorithm	Accuracy	Sensitivity	Specificity	AUC	*p*-Value
XGBoost	0.690	0.684	0.693	0.738	-
LightGBM	0.682	0.682	0.682	0.735	0.097
Random forest	0.670	0.670	0.670	0.724	<0.001
MLP	0.633	0.632	0.633	0.667	<0.001
Logistic regression	0.611	0.611	0.611	0.634	<0.001

Note. (1) The DeLong test was utilized for significance testing. (2) The LightGBM model does not exhibit significant differences compared with the XGBoost model, whereas notable differences are observed with other models, with the XGBoost model demonstrating superior quality.

**Table 5:** Performance comparison between all-feature model and significant-feature model.

Model	Accuracy	Sensitivity	Specificity	AUC	DeLong Test (*p*-Value)
All-feature model (18 features)	0.690	0.684	0.693	0.738	-
Significant-feature model (8 features)	0.678	0.679	0.678	0.734	0.058

Note: The 8 features utilized were length of illness, age, exercise, average meals per day, cho. total, TGs, glucose AC, and HbA1c.

## Data Availability

The original contributions presented in this study are included in this article. Further inquiries can be directed to the corresponding authors.
